# Iodinated Contrast Media in Oncologic CT: A Narrative Review of Safety, Risk Stratification, and Practical Considerations

**DOI:** 10.3390/diagnostics16101507

**Published:** 2026-05-15

**Authors:** Sabina-Oana Vasii, Florin-Gabriel Crișan, Sandra-Monica Lazăr, Claudiu Ioniță, Dan Iliescu, Ioana Ioniță, Daniel-Claudiu Malița, Mirela Voicu, Adrian Voicu, Lucreția Udrescu

**Affiliations:** 1 Center for Drug Data Analysis, Cheminformatics, and the Internet of Medical Things, Victor Babeş University of Medicine and Pharmacy Timişoara, 300041 Timişoara, Romania; sabina.vasii@umft.ro; 2 Doctoral School, Victor Babeş University of Medicine and Pharmacy Timişoara, 300041 Timişoara, Romania; florin.crisan@umft.ro; 3 First Department of Internal Medicine, Victor Babeş University of Medicine and Pharmacy Timişoara, 300041 Timişoara, Romania; sandra.lazar@umft.ro (S.-M.L.); ionita.ioana@umft.ro (I.I.); 4 Department of Hematology, Emergency Municipal Hospital, 300254 Timişoara, Romania; 5 Department IX–Surgery I, Discipline Surgical Semiology I and Thoracic Surgery, Victor Babeş University of Medicine and Pharmacy Timişoara, 300041 Timişoara, Romania; claudiu.ionita@umft.ro (C.I.); dan.iliescu@umft.ro (D.I.); 6 Department of General Surgery I, Emergency Municipal Hospital, 300254 Timişoara, Romania; 7 Multidisciplinary Research Center for Malignant Hemopathies, Victor Babeş University of Medicine and Pharmacy Timişoara, 300041 Timişoara, Romania; 8 Department XV-Orthopedics-Traumatology, Urology, Radiology and Medical Imaging, Victor Babeş University of Medicine and Pharmacy Timişoara, 300041 Timişoara, Romania; malita.daniel@umft.ro; 9 Department II–Pharmacology, Physiology and Physiopathology, Victor Babeş University of Medicine and Pharmacy Timişoara, 300041 Timişoara, Romania; adrian.voicu@umft.ro; 10 Research Center for Experimental Pharmacology and Drug Design (X-Pharm Design), Victor Babeş University of Medicine and Pharmacy Timişoara, 300041 Timişoara, Romania; 11 Department I–Clinical Pharmacy and Drug Analysis, Victor Babeş University of Medicine and Pharmacy Timişoara, 300041 Timişoara, Romania

**Keywords:** post-contrast acute kidney injury, contrast-induced nephropathy, hypersensitivity reactions, immune checkpoint inhibitors, thyroid dysfunction, differentiated thyroid carcinoma, drug interactions

## Abstract

**Background:** Iodinated contrast media are essential for oncologic imaging but raise specific safety concerns because cancer patients are often exposed to repeated contrast-enhanced computed tomography, nephrotoxic drugs, immune-modulating therapies, and, in selected cases, radioiodine-dependent diagnostic or therapeutic pathways. **Methods:** We performed a narrative review based on an exploratory search followed by a focused search targeting iodinated contrast use in oncology-related settings. Studies were included if they addressed renal risk and post-contrast acute kidney injury, hypersensitivity and acute adverse reactions, or thyroid dysfunction with radioiodine-related implications. We also considered clinically relevant studies on drug interactions, isotope studies, and laboratory confounding. **Results:** The evidence base was methodologically heterogeneous, with renal safety as the predominant domain. Kidney injury after contrast-enhanced imaging in cancer patients appeared frequently multifactorial, supporting the broader concept of post-contrast acute kidney injury rather than automatic attribution to contrast alone. Hypersensitivity reactions to modern nonionic iodinated contrast media were generally uncommon, with severe reactions rare, although immune-modulating therapies may alter risk. Thyroid-related effects were usually transient but relevant in patients with thyroid autonomy and in differentiated thyroid carcinoma, where contrast exposure may affect scintigraphy and radioiodine planning. **Conclusions:** In oncology, iodinated contrast use requires individualized, field-specific risk stratification instead of reflexive avoidance.

## 1. Introduction

Contrast-enhanced computed tomography (CT) is central to modern cancer care because it provides detailed and high-resolution images and supports initial staging, treatment planning, response assessment, evaluation of complications, and longitudinal surveillance [1,2]. In many patients, these examinations are repeated across the course of disease, which means that decisions regarding iodinated contrast media are rarely isolated radiologic choices and are instead part of a broader oncologic management strategy. This context is particularly relevant because patients with cancer often present with renal vulnerability, multimorbidity, and exposure to potentially nephrotoxic systemic therapies. In some settings, especially onco-hematology, treatment complexity and polypharmacy further increase the likelihood of adverse effects, competing causes of organ dysfunction, and clinically relevant drug-related confounding [3,4,5,6,7,8].

The safety of iodinated contrast media in oncology cannot be reduced to a single toxicity model. Modern nonionic iodinated contrast agents are widely used because they improve diagnostic performance and generally have favorable pharmacologic profiles, yet their administration may still be associated with acute kidney injury, hypersensitivity reactions, and, in selected contexts, clinically relevant thyroid effects [9,10,11,12]. Experimental and mechanistic studies have also shown that iodinated contrast media may influence endothelial function, medullary oxygenation, oxidative stress, and blood rheology, providing biologically plausible pathways for adverse effects in susceptible patients, even if the clinical expression of these mechanisms depends strongly on baseline risk and concomitant exposures [13,14,15,16,17].

In oncology, the interpretation of contrast-related risk is further complicated by concomitant medication use. Patients frequently receive combinations of cytotoxic chemotherapy, targeted agents, corticosteroids, anti-infective drugs, and supportive treatments, all of which may alter renal risk, interact pharmacodynamically or pharmacokinetically with other therapies, or complicate attribution of post-imaging adverse events [6,7,8,18,19,20,21,22]. Although iodinated contrast media are not conventionally discussed as major perpetrators of classic drug-drug interactions, they may still create clinically important problems through coexposure with nephrotoxic agents, interference with selected laboratory tests, and effects on isotope-based diagnostic or therapeutic pathways [18].

The thyroid is a significant concern in oncology because exposure to iodinated contrast media can lead to transient thyroid dysfunction in vulnerable patients. This exposure may also interfere with diagnostic scintigraphy or planning for radioactive iodine treatment in cases of differentiated thyroid carcinoma [23,24,25]. Additionally, many patients undergoing imaging with iodinated contrast media are already part of a complex therapeutic and diagnostic framework that includes polypharmacy, potential interference from isotope studies, and various laboratory confounding factors—all of these can complicate the attribution and management of any adverse events that arise [26,27,28,29,30].

Against this background, a clinically focused narrative review is appropriate because it allows integration of heterogeneous evidence and emphasizes issues that are directly relevant to oncology practice. In this context, we designed a search-informed narrative review to examine the use of iodinated contrast media in oncology, focusing on the three main domains identified above and also considering clinically relevant data on drug interactions and laboratory confounding. We aimed to provide a practical framework for interpreting contrast-related risk in cancer care and to support individualized decision-making rather than reflexive contrast avoidance.

Because iodinated contrast media are primarily relevant to contrast-enhanced CT, our review emphasizes how safety issues related to contrast media influence the clinical application of CT in cancer staging, response assessment, and surveillance.

## 2. Methods

We conducted a search-informed narrative review of iodinated contrast media in oncology treatment settings using PubMed as the bibliographic source. The search covered articles published from January 2000 to December 2025. We applied a two-stage search strategy: an exploratory search designed to create a broad screening library of clinically relevant contrast-media literature, followed by a focused search aimed at identifying studies specifically addressing iodinated contrast in cancer contexts. The search combined MeSH and free-text terms related to iodinated contrast media, oncologic imaging, renal safety, hypersensitivity reactions, thyroid dysfunction, drug interactions, isotope studies, and laboratory interference. Representative terms included *contrast media*, *iodinated contrast*, *contrast-enhanced CT*, *cancer*, *chemotherapy*, *immunotherapy*, *immune checkpoint inhibitors*, *radioiodine*, *thyroid carcinoma*, *contrast media drug interactions*, *isotope studies*, and *laboratory interference*, combined using Boolean operators AND/OR.

We retained studies that directly contributed to one of the predefined clinical domains: renal risk and post-contrast acute kidney injury, hypersensitivity and acute adverse reactions, or thyroid dysfunction and radioiodine-related implications, or drug interactions, isotope studies, and laboratory confounding in CT contrast pathways. We included clinical cohorts, systematic reviews, narrative or practice-oriented reviews, consensus or guideline-type articles, mechanistic studies with clinical relevance, and selected case reports when they illustrated clinically important emerging phenomena, particularly in oncology-specific settings. We excluded studies focusing only on gadolinium-based MRI contrast agents, non-iodinated contrast agents, imaging contexts unrelated to CT or iodinated contrast media, or lacking clinically relevant information on contrast-media safety, risk stratification, or management.

For each study in our review, we extracted the study design, oncology setting, treatment context, contrast-related issue, and main clinically relevant findings. Instead of using quantitative methods, we synthesized the literature through thematic analysis. We evaluated the evidence based on its relevance to oncology, methodological strength, biological plausibility, and practical clinical value.

## 3. Renal Safety of Iodinated Contrast Media in Oncologic CT

Renal safety remains the most extensively discussed domain in the literature on iodinated contrast media in oncology, which is clinically justified [31]. Patients with cancer often require multiple contrast-enhanced CT scans across staging, treatment monitoring, and surveillance. However, many such patients already have a high renal vulnerability related to chronic kidney disease, older age, dehydration, heart failure, sepsis, and exposure to nephrotoxic drugs [32,33,34,35,36,37,38,39]. In this context, a rise in serum creatinine after imaging is common, but its attribution to contrast alone is often problematic [40,41]. Oncology clinicians must cautiously interpret renal injury following contrast administration, considering it within the broader context of multifactorial post-contrast acute kidney injury, instead of attributing it solely to the toxic effects of the contrast agent [1,31].

Table 1 summarizes the studies addressing renal issues related to iodinated contrast media in oncology treatment settings.

A major conceptual advance in recent years has been the distinction between contrast-induced acute kidney injury (CI-AKI) and post-contrast acute kidney injury (PC-AKI). CI-AKI implies a causal relationship between the administration of contrast agent and the deterioration of renal function, whereas PC-AKI describes kidney injury observed after contrast exposure, regardless of whether contrast is the true cause. This distinction is particularly relevant in cancer patients, in whom coincident renal insults are common. The onco-nephrology white paper by Cosmai et al. [1] is especially useful in this regard, because it explicitly recommends the use of PC-AKI when causality cannot be established and emphasizes that AKI in oncology is frequently multifactorial [1,43]. Traditionally, contrast-induced nephropathy has been defined as an increase in serum creatinine of at least 25% from baseline or an absolute increase of 0.5 mg/dL, usually within 48–72 h after intravascular iodinated contrast administration. However, this definition is based on temporal association and does not always establish causality. In this review, we define PC-AKI as renal dysfunction occurring in temporal association with contrast administration, without confirmation of a direct causal relationship. The term CI-AKI is reserved for instances where the literature explicitly identifies or discusses a causal link between contrast media exposure and kidney injury [1,49,50].

A strong oncology cohort in this set is the study by Latcha et al., which compared contrast-enhanced and non-contrast CT examinations in a large cancer population. Although the crude AKI rate was 7.3% after contrast-enhanced CT and 11.4% after non-contrast CT, the key finding was not a protective effect of contrast omission, and the AKI risk was driven predominantly by baseline renal impairment and patient-related vulnerability. Lower estimated glomerular filtration rate (eGFR), recent chemotherapy, congestive heart failure, and prior AKI were associated with a higher risk, whereas contrast exposure itself was not an independent signal after propensity adjustment. This finding is important as it argues against the tendency to attribute every post-CT creatinine rise in oncology to intravenous iodinated contrast [42].

Werner et al. further investigated a higher-risk subgroup of oncology patients with baseline eGFR below 60 mL/min/1.73 m^2^ who underwent contrast-enhanced CT with a reduced dose of iodixanol. In this cohort, overall PC-AKI was 6.3%. When alternative causes of renal deterioration were excluded, the presumed CI-AKI rate dropped to 3.8%. No patient required specific treatment for AKI, and there was no documented permanent post-contrast renal worsening. Although the study is retrospective and lacks a comparator arm, it offers a valuable insight: even in higher-risk oncology patients with chronic kidney disease, contrast-enhanced CT is not universally prohibitive when performed using cautious protocols and risk-aware supportive care [43].

A recent work by Andreucci et al. highlights the need to reconsider renal contrast toxicity, presenting evidence for a model of renal injury that includes factors like renal vasoconstriction, reduced medullary oxygenation, and oxidative stress. Their review is valuable because it captures the present uncertainty in the field: epidemiologic studies have challenged the magnitude of CI-AKI after intravenous contrast, but experimental work continues to support the plausibility of true contrast-mediated renal injury in susceptible kidneys [12].

From a clinical oncology perspective, Heiken’s review explains why cancer patients are a distinct risk group. Renal impairment is common in patients with malignancy, and the usual risk factors for contrast nephropathy are amplified by age, dehydration, diabetes, and concomitant nephrotoxic therapies. Heiken also highlighted that using intravenous iodinated contrast for CT generally poses a lower renal risk compared to intra-arterial administration; at-risk patients should not automatically be denied contrast-enhanced CT if the expected diagnostic or oncologic benefit outweighs the renal risk. Instead, screening and precautionary measures should be incorporated into routine oncologic imaging practice [31].

This prevention-oriented approach is further supported by Grassedonio et al., who reviewed mechanisms, risk factors, and preventive strategies for PC-AKI in patients with cancer undergoing contrast-enhanced CT. Their review is clinically useful because it translates the oncology-specific risk framework into practical measures, including baseline renal function assessment, identification of high-risk patients using recently proposed risk scores, intravenous saline hydration, and preferential use of low-osmolar or iso-osmolar contrast media in selected vulnerable individuals. Instead of supporting reflexive contrast avoidance, this work advocates structured prevention protocols tailored to the patient’s renal and oncologic risk profile [44].

Pre-procedural management should prioritize the identification of patients at clinically meaningful renal risk and correction of modifiable factors prior to contrast-enhanced CT. Particular attention is warranted for patients with reduced eGFR, recent acute kidney injury, dehydration, heart failure, sepsis or acute infection, repeated recent contrast exposure, or concomitant use of nephrotoxic anticancer or supportive care drugs [1,31,44,49,50]. Hydration remains the primary preventive measure for patients at increased risk. Intravenous isotonic saline is generally preferred for higher-risk patients when clinically feasible, while oral hydration may be suitable for lower-risk outpatients who can reliably maintain fluid intake [44,49,50]. Unnecessary prolonged fasting should be avoided, as it may exacerbate dehydration, particularly in frail oncology patients, older adults, individuals with poor oral intake, and those undergoing imaging during hot seasons. When fasting or dietary restriction is necessary, patients should receive clear instructions regarding permitted fluid intake and the timing of imaging [49,50].

Outpatient follow-up should be risk-adapted. In low-risk patients with stable renal function, routine post-contrast creatinine testing is unlikely to be necessary in most settings, and mild transient renal changes may resolve without detection. However, in patients with eGFR < 30 mL/min/1.73 m^2^, recent AKI, acute illness, dehydration, ongoing nephrotoxic therapy, or other clinically meaningful renal vulnerability, serum creatinine reassessment within 48–72 h after contrast exposure may help detect PC-AKI and guide management [1,44,49,50].

Repeated contrast exposure may also be relevant in selected surveillance populations. Using a nationwide database, Koo et al. examined patients with early gastric cancer undergoing repetitive contrast-enhanced CT after curative treatment and found that greater cumulative contrast-enhanced CT exposure was associated with subsequent chronic kidney disease. Although this cohort was highly specific and excluded several major competing renal risk factors, the study is relevant because it suggests that the long-term renal trajectory may deserve attention in patients who undergo frequent serial imaging, even when the risk associated with a single examination appears limited [45].

A further challenge in oncology is that even the assessment of kidney function is often unstable or misleading. Malyszko et al. argue that renal evaluation in cancer should not be reduced to a purely glomerulocentric interpretation of serum creatinine or estimated GFR, because both overestimation and underestimation of renal function can alter treatment decisions. Their review emphasized that kidney dysfunction in oncology may involve glomerular, tubular, and vascular factors, and that inaccurate renal estimation may lead to incorrect dosing or unnecessary treatment exclusions [46].

In pediatric and young adult hemato-oncology patients, Soeorg et al. showed that commonly used eGFR equations and simplified measured-GFR formulas overestimated renal function when compared with iohexol clearance modeled using a three-compartment pharmacokinetic approach [47]. In a different context, Topletz-Erickson et al. showed that tucatinib can increase serum creatinine by inhibiting renal transporters without affecting iohexol-measured GFR or cystatin C-based eGFR; this finding illustrates how apparent decline in renal function in oncology may sometimes be attributed to altered tubular handling of creatinine rather than true loss of filtration. Although these studies do not specifically focus on contrast-induced nephrotoxicity, they are valuable in our review because they clarify why renal risk attribution in oncology is often more difficult than a single creatinine value suggests [48].

Complementing these data, the 2025 Acute Disease Quality Initiative consensus by Renaghan et al. provides updated guidance on the nephrotoxic effects of anti-cancer therapies in general. This report reinforces the multifactorial nature of AKI in oncology; these factors include contrast media, chemotherapy, targeted agents, immunotherapy, dehydration, and sepsis. It further supports the PC-AKI framework instead of attributing it solely to iodinated contrast. These contributions strengthen the case for individualized prevention protocols and ongoing renal surveillance rather than reflexive contrast restriction [37].

## 4. Hypersensitivity and Acute Reactions in Contrast-Enhanced CT

Hypersensitivity and other acute adverse reactions to iodinated contrast media represent a second major safety domain in oncology, distinct from renal toxicity [51,52,53]. Hypersensitivity reactions are less frequent, most are mild, only a small minority are potentially life-threatening, and delayed cutaneous reactions may be underrecognized because they may occur after the patient has left the imaging unit [54,55,56,57,58,59]. Hypersensitivity reactions are immediate and nonimmediate. Immediate reactions occur within 1 h after contrast administration, and nonimmediate reactions begin from 1 h up to 10 days later. Immediate reactions may resemble anaphylaxis, whereas delayed reactions are usually cutaneous, most often maculopapular exanthema [60,61]. Severe and fatal events are rare, but they remain clinically relevant because they are not completely predictable [53,62,63,64].

The literature on delayed reactions is heterogeneous. Although nonimmediate hypersensitivity reactions are generally defined as those beginning more than 1 h after contrast administration and occurring up to several days later, individual studies differ in the specific time windows used, methods of case identification, degree of dermatologic confirmation, and application of diagnostic tests such as patch testing, delayed-reading intradermal testing, lymphocyte transformation testing, or biopsy. Causality assessment also varies across studies, particularly when re-exposure data or formal allergy work-up are unavailable. Consequently, reported rates and phenotypes of delayed reactions should not be considered fully interchangeable across studies [54,57,58,59,61,62,65].

This limitation is especially relevant in oncology, where delayed rashes, fever, eosinophilia, or systemic inflammatory manifestations may also be caused by immune checkpoint inhibitors, targeted therapies, cytotoxic chemotherapy, anti-infective drugs, viral infections, or supportive drugs [51,66,67,68]. In this setting, temporal association with contrast-enhanced CT may identify a post-contrast event, but it does not necessarily establish contrast causality unless timing, morphology, culprit agent, alternative causes, and diagnostic testing are carefully documented [54,57,58,61,62].

Given that the evidence regarding iodinated contrast hypersensitivity encompasses both established general radiology literature and a more recent oncology-specific body of work, the studies included in this section are organized into two thematic groups. Table 2 distinguishes between the general hypersensitivity framework and oncology-specific immune-modulation studies.

The overall frequency of acute reactions to modern nonionic iodinated contrast media appears low. In the meta-analysis by Suh et al., which included 30 studies and 1,360,488 exposures, the pooled incidence of overall acute adverse reactions was 1.03%, whereas the pooled incidence of severe reactions was 0.0141%. Although reaction rates differed between individual nonionic agents, the differences were not statistically significant after adjustment for confounders such as study design and premedication practices, thus arguing against simplistic claims that one modern nonionic agent is safer than another with respect to acute hypersensitivity [11].

Large cohort data also show that risk is prevalent in selected patient groups. In the study by Li et al., based on 120,822 enhanced CT examinations, the incidence of adverse drug reactions was 0.4% in patients with underlying diseases and 0.44% in those without, indicating no major overall excess risk simply from the presence of comorbidity. Instead, risk was highest in patients with asthma, cardiac insufficiency, and especially previous reactions to iodinated contrast, in whom the reaction rate reached 7.17%. Reactions were also more common at higher injection doses and speeds. These findings support a more selective model of screening and preparation, focused on prior reactors and specific high-risk phenotypes instead of broadly excluding all medically complex patients [10].

The current understanding suggests that contrast reactions are not solely anaphylactoid or non-specific; Brockow and Idée emphasize that the pathophysiology is heterogeneous [62,63]. Immediate reactions may involve non-specific mediator release, complement activation, bradykinin-related pathways, or, in at least some cases, true IgE-mediated allergy. Various diagnostic methods, such as patch testing, intradermal testing, lymphocyte transformation studies, and biopsy findings, support a T-cell-associated process in at least a subset of patients, suggesting that delayed reactions are mediated, at least in part, by an immunologic mechanism. Idée also notes that delayed reactions are more frequent in interleukin-2-treated patients, an observation that is especially relevant in oncology because it implies that the host immune state can modify contrast tolerance [63].

This mechanistic heterogeneity has practical implications for diagnosis and prevention. Brockow argues that skin testing can support the diagnosis of both immediate and delayed hypersensitivity, particularly when the clinical history is convincing; however, cross-reactivity between different contrast agents is common, and a negative skin test does not rule out clinical reactivity [62]. Furthermore, the available literature does not support overconfidence in pharmacologic prophylaxis. Idée concludes that no prophylactic regimen has shown undeniable efficacy, and Brockow notes that severe repeated nonimmediate reactions occurred, even with corticosteroid premedication [63]. Hence, these studies support a cautious strategy based on precise reaction phenotyping, documentation of the culprit agent when possible, and individualized planning instead of reliance on vague labels or routine prophylaxis.

A particularly important practical problem is the so-called iodine allergy. Böhm et al. showed that this label was unspecified in 84.3% of cases and was associated with substantially poorer documentation of previous symptoms than more precise contrast-related diagnoses. This lack of clarity had further clinical implications: the proportion of unenhanced CT examinations was highest in the iodine allergy group, and adverse drug reactions after prophylactic management occurred only in that group [69]. This message has direct relevance for oncology imaging, where inaccurate allergy information may influence staging and response assessment; accordingly, the nonspecific iodine allergy should be replaced with proper identification of the culprit agent and characterization of the reaction.

Although most contrast reactions are mild, the severe end of the spectrum remains clinically important because screening does not identify every high-risk patient. Palmiere Bonetti’s forensic review of fatal anaphylaxis due to contrast media found that fatalities are extremely rare, but that only a minority of fatal cases had the classic identifiable predisposing factors or known prior exposure. In the medicolegal cases examined by the authors, mast cell tryptase and total IgE were increased in all subjects studied. This article highlights that while known risk factors are important in prevention, they do not completely rule out catastrophic reactions in apparently low-risk patients [70].

### Hypersensitivity in the Context of Oncological Immunomodulation

The oncology literature adds an important newer layer to this field: contrast tolerance may be altered by immune-modulating anticancer therapy. Ridolfi et al. retrospectively evaluated 3521 cancer patients undergoing contrast-enhanced CT after systemic treatment and found that immediate contrast-related adverse reactions occurred in 12% of patients treated with ipilimumab, 5% of those treated with cytokines, and 2% of those receiving chemotherapy or targeted therapy. In the ipilimumab and cytokine groups, the allergic events predominated. Although the study is retrospective and the immunotherapy cohorts were small, it supports the clinically important hypothesis that immune activation, particularly CTLA-4 blockade, may increase the frequency of immediate allergic-like reactions to iodinated contrast agents [51].

Hammond et al. described a patient with metastatic renal cell carcinoma who had previously tolerated more than 20 contrast-enhanced CT examinations with amidotrizoate, but after treatment with atezolizumab developed a sequence of increasingly severe cutaneous reactions culminating in Stevens–Johnson syndrome. Re-exposure reproduced the syndrome, providing compelling evidence that the contrast agent was responsible, and not merely the checkpoint inhibitor by itself; laboratory analysis demonstrated amidotrizoate-responsive T-cell clones secreting IFN-γ, granzyme B, and perforin. The authors interpret this as evidence that checkpoint inhibition may shift a previously tolerated antigen from immune tolerance toward pathogenic recall activation [66]. Hence, it is important to note that some immune-related adverse events after contrast exposure may reflect an interaction between the contrast agent and an immunologically primed host rather than a de novo reaction to one drug in isolation.

The same principle may extend to rare cardiovascular phenotypes of contrast hypersensitivity. Zmolik et al. reported a case of a woman with bronchial adenocarcinoma receiving pembrolizumab who developed Type 1 Kounis syndrome after administration of iopromide, with acute chest pain, ischemic ECG changes, angiographically documented coronary vasospasm, and subsequent delayed rash [71]. As a single case, this report cannot inform incidence, but it illustrates the broad range of clinically relevant contrast hypersensitivity in the immunotherapy era beyond urticaria and exanthema to include allergic acute coronary syndromes.

The hypersensitivity literature supports a broader and more nuanced interpretation of iodinated contrast safety in oncology. Acute reactions to modern nonionic contrast media are uncommon, and severe reactions are rare, but the risk is concentrated in selected patients, especially those with previous reactions and certain cardiopulmonary or allergic backgrounds. Most importantly for oncology, immune-modulating therapies such as interleukin-2 and checkpoint inhibitors appear capable of altering reaction susceptibility or phenotype. In practical terms, this means that contrast safety in cancer care should rely on precise history-taking, correct labeling, awareness of the immune context, and preparation for immediate and delayed events [51,66,70].

## 5. Thyroid Dysfunction and Radioiodine-Related Implications After Contrast-Enhanced CT

The impact of iodinated contrast media on the thyroid is a unique safety concern in oncology, while renal injury and hypersensitivity are typically associated with acute toxicity, thyroid effects arise from the iodine load during imaging that may become relevant over days to weeks after exposure [26,27,28,29,30]. These effects are relevant for patients with pre-existing thyroid issues (e.g., nodular or autonomous thyroid disease) and those with differentiated thyroid carcinoma (DTC), as iodine exposure may interfere with thyroid scintigraphy or radioiodine treatments [23,24,25].

Table 3 summarizes the studies included in the thyroid domain of this review.

A practice-oriented review reports that clinically relevant iodine-induced thyroid dysfunction is generally rare, but that risk is concentrated in older patients with autonomous nodular thyroid disease and cardiac comorbidity; the same review also supports caution regarding recent exposure to iodine before scintigraphy or radioiodine therapy [75].

More recent prospective data show that short-term thyroid changes after iodinated contrast are often mild and transient rather than persistently clinically significant. In a multicentre prospective cohort study by Peng et al., 200 adults received IV iodinated contrast and underwent thyroid function and urinary iodine assessment before exposure and then at 1 week and 1 month, with follow-up at 3 months for those who developed abnormalities. Urinary iodine peaked at 1 week, patients with thyroid nodules had a higher risk of subclinical hyperthyroidism at 1 month, and most newly developed abnormalities had largely returned to normal by 3 months without intervention. Therefore, this study highlights that iodinated contrast can perturb thyroid function measurably, but in many patients, the effect is self-limited and not permanently disruptive [23]. Despite its relevance, this study should be interpreted cautiously in oncology because the investigators explicitly excluded patients with malignant tumors, thyroid cancer, immune checkpoint inhibitor exposure, tyrosine kinase inhibitors, interferon, and several other thyroid-active therapies.

The translational study by Vassaux et al. challenges the assumption that impaired thyroid radioiodine uptake after contrast is explained only by free iodide contamination. Using in vitro systems, murine imaging, human scintigraphy, and tissue analyses, the authors showed that iodinated contrast media reduced thyroid radiotracer uptake while producing a marked reduction in sodium-iodide symporter (NIS) expression in thyrocytes; by contrast, salivary-gland uptake and NIS expression were not similarly affected. They concluded that contrast agents can perturb thyroid iodide uptake independently of free iodide and that this effect may be thyroid-selective [73]. This article offers a more sophisticated mechanistic explanation for why thyroid radioiodine handling may remain abnormal even after simple measures of iodine exposure begin to normalize.

In differentiated thyroid carcinoma (DTC), contrast-enhanced CT is often clinically useful, particularly for large, invasive, or nodal disease, although iodine load may delay postoperative radioiodine ablation. Mishra et al. addressed this question in a prospective controlled study including four groups of patients, among them DTC patients undergoing preoperative contrast-enhanced CT and DTC patients managed without contrast exposure. Urinary iodine concentrations were significantly higher shortly after contrast administration. However, these differences were no longer significant at follow-up 4 to 6 weeks later, and the authors concluded that preoperative contrast-enhanced CT with non-lipophilic contrast did not result in long-term iodine retention. They also argued that 6 weeks after total thyroidectomy appeared to be a reasonably safe interval to proceed with radioiodine evaluation or treatment, indicating a need to reassess older recommendations to delay for up to 3 months [74].

Mishra and Vassaux highlight an important tension in the literature. On the one hand, conservative guidance on endocrine and nuclear-medicine favors caution, selective monitoring, and delay of scintigraphy or radioiodine therapy after major iodine exposure, especially in patients at high risk of iodine-induced hyperthyroidism. On the other hand, more recent prospective and mechanistic work suggests that the problem may be more nuanced than the older iodine washout model implies. Urinary iodine can normalize relatively quickly in many patients, especially after thyroidectomy, and recovery of thyroid radioiodine uptake may not depend only on the elimination of free iodine, because contrast media can also directly alter NIS-mediated uptake. This indicates that avoiding contrast-enhanced CT and delaying radioiodine procedures may not be optimal for all thyroid cancer pathways [73,74].

The practical implication for oncology practice is that thyroid-related risk after iodinated contrast should be individualized. The literature supports a balanced approach between clinically necessary contrast-enhanced imaging that should not automatically be withheld and the risk of short-term thyroid dysfunction and interference with radioiodine administration, particularly in high-risk endocrine phenotypes [73,74,75].

## 6. Drug Interactions, Isotope Studies, and Laboratory Confounding in CT Contrast Pathways

The administration of iodinated contrast agents also requires an approach within a broader pharmacological and diagnostic context, particularly relevant in oncology, where patients typically receive multiple concomitant drugs for cancer, supportive care, and comorbid diseases [67,68,76,77,78,79].

Table 4 summarizes the studies included in this section. In contrast to the previous renal, hypersensitivity, and thyroid sections, this body of literature is smaller and more practice-oriented, with the strongest evidence coming from guideline-style reviews and clinical pharmacology studies and less from comparative oncology cohorts.

In a prevalence study of ambulatory patients receiving intravenous anticancer therapy, van Leeuwen et al. found that 58% had at least one potential drug interaction, 34% of DDIs were major, and 11% involved over-the-counter drugs. This polypharmacy background means that contrast-related decisions in oncology rarely occur in isolation and may be influenced by competing drug toxicities, altered attribution of events, and the practical management of concurrent medication [7].

A dedicated framework for this topic is the ESUR-based review by Morcos et al., which explicitly organizes DDIs involving contrast media together with other drugs into clinically relevant groups. These groups include drugs involved in contrast-related renal impairment, drugs that enhance the renal effects of contrast media, drugs that increase allergy-like reactions, drugs that interfere with hematologic or cardiac effects of contrast media, interference with isotope studies, incompatibility when contrast is mixed with other agents, and interference with biochemical assays [18]. This framework is particularly valuable because it shows that contrast safety is not limited to direct toxicity of the imaging agent itself, but also involves the surrounding drugs and diagnostic environment.

A first clinically important interaction pathway involves drug retention secondary to deterioration in renal function. Morcos et al. identify metformin as the clearest example: if contrast-induced renal dysfunction occurs, metformin may accumulate and increase the risk of lactic acidosis. The same review also notes that NSAIDs, aminoglycosides, cyclosporine, and cisplatin may augment the nephrotoxic effects of contrast media, whereas diuretics may worsen dehydration and thereby increase the risk of post-contrast renal injury. In oncology, this issue is of particular relevance because of nephrotoxic drugs that may blur the distinction between contrast-associated injury and background treatment-related renal decline [18].

A second interaction pathway concerns hypersensitivity management. According to Morcos et al., patients on beta-blockers, interleukin-2, or interferons have an increased tendency to develop allergy-like reactions after contrast administration. The clinical significance of beta-blockers lies in their potential to reduce the effectiveness of adrenaline and other sympathomimetic drugs when treating severe reactions. The same review highlights that delayed reactions are more likely in patients treated with interleukin-2, an observation that is especially relevant to oncology because it supports the broader concept that the immune context of the host can influence tolerance to contrast agents [18].

In clinical practice, there is a persistent but incompletely resolved question of drugs that should be withheld before contrast procedures. Hiremath et al. show that this uncertainty has been recognized explicitly in the literature. Their systematic review shows that many clinical guidelines recommend withholding renin–angiotensin system blockers, NSAIDs, diuretics, and metformin before contrast administration in patients with kidney disease, diabetes, or cardiovascular disease. It also emphasizes that the supporting evidence is limited and that drug discontinuation itself may lead to adverse consequences or failure to restart treatment [80].

Contrast media may also interfere with isotope studies, which is especially important because the thyroid domain intersects directly with nuclear medicine pathways [25,28,85,86]. Morcos et al. recommend avoiding administration of contrast media for at least 24 h before isotope studies involving bone imaging or red blood cell labeling, and they also highlight the need to consider thyroid-specific effects when radioiodine-based investigations or therapies are planned [18].

Contrast media may also interfere with biochemical assays and thereby complicate the interpretation of laboratory data obtained shortly after imaging [25,28,82,83,84]. Morcos et al. note that iodinated contrast media may affect measurements of bilirubin, copper, iron, phosphate, and proteins in blood, and recommend that non-emergency biochemical analyses be performed before contrast injection or delayed for at least 24 h, with even greater caution in patients with renal impairment [18]. Therefore, the unexpected laboratory abnormalities immediately after contrast exposure should not automatically be accepted at face value, but should be interpreted in light of recent contrast administration and, when necessary, discussed with the laboratory.

## 7. Discussion

The literature review emphasizes that contrast media in oncology should be viewed beyond a mere list of adverse effects. Three consistent domains emerge from the available evidence: renal safety, hypersensitivity and acute adverse reactions, and thyroid dysfunction affecting radioiodine-based management. A fourth, smaller but still relevant body of literature indicates that the administration of contrast agents should also be interpreted within a broader environment of concomitant drugs, isotope studies, and laboratory testing. These findings suggest that contrast-related risk in oncology is not uniform, being concentrated in selected vulnerable patients and strongly influenced by the surrounding treatment context [1,11,18,74].

The renal domain remains the most extended area of evidence, but it is also the one in which overattribution is most likely. The major strength of the oncology-specific renal literature is that it shifts the discussion away from a simplistic assumption that any rise in creatinine after contrast-enhanced CT is necessarily contrast-induced. Hence, PC-AKI is often the more appropriate term in oncology, where causality is difficult to establish because multiple nephrotoxic exposures and competing renal injuries commonly coexist [1,42,43].

At the same time, the reviewed literature does not justify dismissing renal toxicity as a purely artifactual construct. The contrast media are nephrotoxic, but their impact largely depends on the host’s susceptibility. In cancer patients, susceptibility is heightened by factors such as chronic kidney disease, dehydration, advanced age, recent chemotherapy, cardiovascular disease, repeated imaging, and the challenges associated with accurately estimating renal function reserve. In this context, the field of oncology does not eliminate contrast nephrotoxicity; instead, it complicates causal interpretation, because post-imaging renal dysfunction often reflects a combined effect of contrast exposure and multiple concurrent renal stressors [1,12,31].

The risk of PC-AKI is context-dependent. Beyond baseline renal function and nephrotoxic co-medication, factors such as hydration status, nutritional intake, infection, hospitalization, access to pre- and post-procedural monitoring, and healthcare delivery pathways may influence the incidence of renal dysfunction following contrast-enhanced CT [1,31,44]. Regional and seasonal variables may further modify risk. For instance, in regions with hot summers or limited access to adequate hydration, dehydration prior to imaging may occur more frequently and increase susceptibility to PC-AKI [31,44]. These contextual factors should be carefully considered when translating published evidence into local clinical practice. From a practical standpoint, renal protection is not limited to contrast-agent choice. It also includes reviewing nephrotoxic co-medication, avoiding unnecessary dehydration or prolonged fasting, selecting oral or intravenous hydration according to risk, and ensuring appropriate follow-up in selected high-risk oncology patients [1,44,49,50].

Hypersensitivity to iodinated contrast media in oncology cannot be reduced to a simplistic binary response regarding a history of contrast allergy. Immediate and delayed reactions vary in timing, phenotype, and mechanism, and severe events are rare. Differences between modern nonionic agents are not significant after adjustment for confounders such as study design and premedication. Thus, contemporary contrast safety relies more on accurate patient risk stratification, thorough documentation, and readiness for acute management rather than a simple ranking of agents [11]. Within that framework, the oncology-specific hypersensitivity literature introduces a newer concept that the host immune state may modify contrast tolerance. Although the literature does not yet justify a new formal risk algorithm, it strongly suggests that some contrast reactions in oncology may emerge from the interaction between contrast exposure and an immunologically primed or checkpoint-disinhibited host rather than from the contrast agent alone [51,66].

Iodinated contrast agents have varying properties that affect tolerability and renal risk. High-osmolar ionic contrast media, which are older formulations, are linked to a higher incidence of adverse reactions and nephrotoxicity compared to modern low-osmolar or iso-osmolar agents. In current CT practice, low-osmolar nonionic contrast media are preferred because they provide effective diagnostic enhancement with better tolerability than high-osmolar ionic agents. Iso-osmolar contrast media, such as iodixanol, may benefit certain patients with impaired renal function; however, comparative data between low-osmolar and iso-osmolar agents remain inconclusive. Systematic reviews and comparative studies show that high-osmolar agents are more nephrotoxic than low- or iso-osmolar agents. A definitive renal safety advantage of iso-osmolar over low-osmolar contrast media has not been established and may vary depending on patient characteristics, route of administration, comparator agent, and the definition of contrast-induced acute kidney injury (CI-AKI) [49,50,87,88,89,90].

For patients with cancer, the key question is not whether all individuals are at high risk for contrast-related renal injury, but whether the oncologic context increases susceptibility or changes the benefit-risk assessment. Factors such as repeated imaging, nephrotoxic systemic therapies, supportive-care medications, infection or sepsis, dehydration, hospitalization, obstructive uropathy, reduced renal reserve, and urgent treatment decisions can influence the evaluation of post-contrast renal dysfunction. Therefore, deviation from standard contrast administration protocols is appropriate when renal risk is clinically significant and modifiable, when the diagnostic benefit is limited, or when alternative imaging can address the clinical question without compromising oncologic management [1,31,37,44,50].

In most oncologic settings outside of thyroid-specific pathways, clinically important thyroid dysfunction following the use of iodinated contrast is likely to be rare and concentrated in selected predisposed patients. Nevertheless, this issue becomes highly relevant in DTC, where recent iodine exposure may affect diagnostic scintigraphy and planning of radioactive iodine therapy. The available studies suggest that older assumptions about prolonged interference may need refinement. However, the thyroid consequences of contrast appear to be both more transient and more mechanistically complex than older dogma implied [73,74].

The cross-cutting literature on interactions and confounding reinforces the need to think of contrast exposure as part of a larger therapeutic ecosystem. Contrast media can interact with other concomitant drugs, especially in cancer patients, complicating treatment and test results. Contrast safety should involve agent selection, reaction prevention, drug review, documentation of recent contrast use in nuclear medicine procedures, and careful interpretation of lab abnormalities post-imaging [7,18].

Our review highlights several limitations in the current evidence base. The literature is methodologically heterogeneous, including retrospective cohorts, narrative and practice-oriented reviews, mechanistic studies, and case reports. Many of the most clinically informative renal studies remain retrospective and are therefore vulnerable to confounding by indication, hydration practices, and selective avoidance of contrast in sicker patients. The literature on oncology-specific hypersensitivity is still limited and relies heavily on data from single centers and illustrative case reports. Studies reporting the impact on thyroid function include clinically useful but relatively specialized studies, and several conclusions relevant to radioiodine timing remain inferential, and they are less derived from large prospective oncology cohorts [42,43,51,74]. Another limitation is that retrospective studies often lack consistent reporting of contrast-agent type, ionicity, osmolality, viscosity, dose, injection parameters, cumulative exposure, repetition frequency, hydration protocols, medication management, and outpatient follow-up. The absence of these variables restricts the ability to determine whether observed renal or hypersensitivity outcomes are due to the contrast agent, patient-specific vulnerability, institutional practices, or unmeasured confounding [50,89,90].

Despite these limitations, several practical conclusions emerge. Iodinated contrast media should not be withheld reflexively in oncology, because contrast-enhanced CT is often indispensable for staging, response evaluation, and surveillance. At the same time, the balance of evidence does not support a uniform high-risk model across all cancer patients. Clinical decision-making should start by assessing the urgency and potential diagnostic value of contrast-enhanced imaging, followed by risk stratification specific to each domain. From a renal perspective, patients with eGFR < 30 mL/min/1.73 m^2^, acute kidney injury, recent renal deterioration, nephrotoxic co-medication, significant comorbidities, or impaired hydration status require particular attention. Risk assessment should also include previous immediate or delayed hypersensitivity reactions, thyroid autonomy or planned radioiodine-based procedures, and potential interference with isotope studies or laboratory testing. Modifiable factors, especially hydration status, unnecessary prolonged fasting, drug review, and documentation of previous contrast reactions, should be optimized before imaging. In this framework, contrast administration should be viewed as a collaborative clinical decision and not just a radiologic event [1,11,18,73].

**Figure 1:**
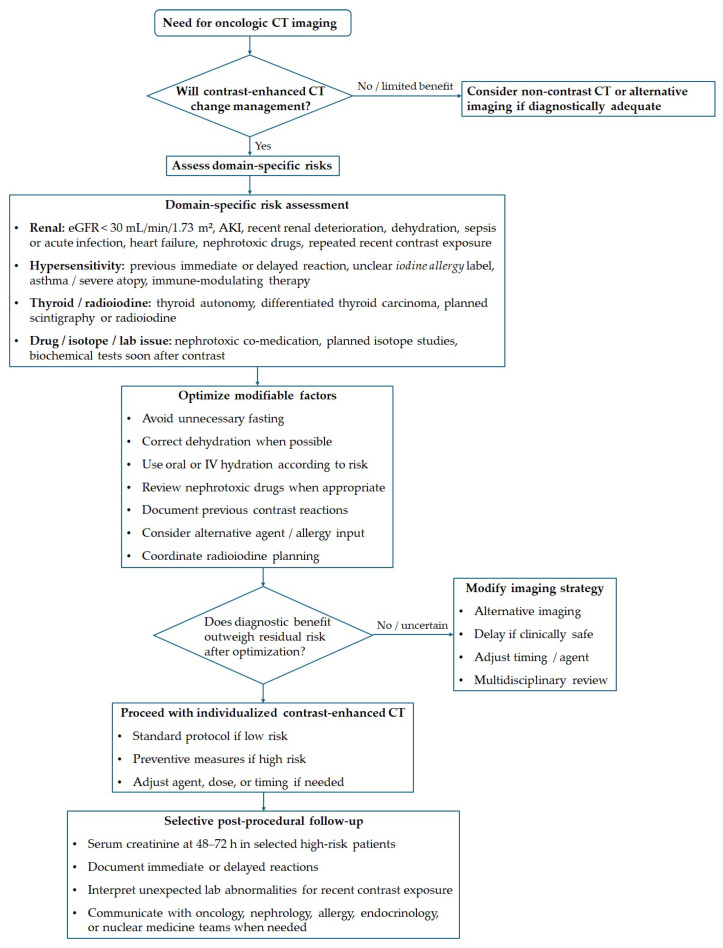
Practical framework for iodinated contrast use in oncologic computed tomography.

Figure 1 illustrates an algorithmic framework for iodinated contrast use in oncologic CT to translate these practical considerations into a clinically applicable structure. The framework starts with assessing the diagnostic benefits of contrast-enhanced imaging, then considers specific risks such as renal vulnerability, hypersensitivity history, thyroid factors, and potential drug or test interferences. It also emphasizes optimizing modifiable factors before imaging, individualizing imaging strategies, and selective post-procedural follow-up for patients with clinically significant risk.

Table 5 summarizes the preventive steps in the framework shown in Figure 1. This table serves as a practical checklist and does not replace local contrast-media protocols, as decisions should reflect institutional policies, available contrast agents, oncology urgency, and patient risk.

Future work should aim to reduce the present uncertainties in a more oncology-specific way. Prospective cohorts are needed to refine the true incidence and predictors of PC-AKI in patients receiving modern systemic therapies, particularly immune checkpoint inhibitors and targeted agents. The hypothesis that immunotherapy modifies contrast hypersensitivity deserves formal prospective evaluation rather than continued reliance on retrospective signal detection and case reports. In thyroid cancers, more data are needed to determine the earliest safe timing of postoperative radioiodine procedures following contrast-enhanced CT; this should ideally integrate both urinary iodine measurements and functional radioiodine uptake endpoints. Last but not least, better separation of true contrast media-induced events from post-contrast events with competing causes would improve both clinical decision-making and the interpretability of future studies [1,51,74].

## 8. Conclusions

The use of iodinated contrast media in oncology is indispensable for accurate imaging assessment. The literature we reviewed here indicates that their safety profile is shaped mainly by three areas of concern, namely kidney vulnerability, hypersensitivity reactions, and thyroid-related consequences in selected patients. The relevance of these risks varies among cancer populations and should be evaluated considering the patient’s comorbidities, ongoing therapies, and future diagnostic or therapeutic plans. Therefore, a personalized multidisciplinary approach is essential to support safe and effective use of contrast agents in cancer care.

## Figures and Tables

**Table 1:** Characteristics of the studies included in the renal safety section.

Study	Evidence Type	Oncology Setting	Treatment Context	Contrast-Related Issue
Latcha et al. [42]	Retrospective clinical cohort	Mixed cancer cohort	Inpatient oncologic imaging; recent chemotherapy and comorbid disease	IV contrast CT versus non-contrast CT; background AKI risk and clinical predictors
Werner et al. [43]	Retrospective clinical cohort	High-risk oncology patients with CKD	Contrast-enhanced CT with reduced-dose iso-osmolar iodixanol	PC-AKI/presumed CI-AKI incidence after IV iodinated contrast in CKD
Andreucci et al. [12]	Narrative/mechanistic review	General clinical population; applicable to oncology risk interpretation	Diagnostic and interventional iodinated contrast exposure	Pathophysiology of CI-AKI, including vasoconstriction, medullary hypoxia, tubular injury, and oxidative stress
Heiken [31]	Narrative review/keynote clinical review	Mixed cancer population	Repeated contrast-enhanced CT in cancer patients, with concurrent nephrotoxic therapies	Oncology-specific CIN risk factors, IV versus intra-arterial risk, and preventive management
Grassedonio et al. [44]	Narrative/practice-oriented review	Mixed oncology	Contrast-enhanced CT in cancer patients with variable renal risk	PC-AKI mechanisms, oncology-specific risk stratification, hydration, and preventive protocols
Koo et al. [45]	Retrospective nested case–control study	Early gastric cancer surveillance cohort	Repeated contrast-enhanced CT after curative treatment	Association between cumulative contrast-enhanced CT exposure and later CKD
Malyszko et al. [46]	Narrative/practice-oriented review	Mixed oncology	Drug dosing, eligibility assessment, and nephrotoxicity monitoring	Limits of creatinine/eGFR alone; renal function assessment in oncology before nephrotoxic exposure
Soeorg et al. [47]	Prospective pharmacokinetic/clinical cohort	Pediatric and young adult hemato-oncology	Renal-function assessment during infection and treatment	Iohexol-based measured GFR versus equation-based overestimation of renal function
Topletz-Erickson et al. [48]	Phase 1 clinical pharmacology plus in vitro transporter study	HER2-positive solid-tumor drug-development context	Targeted therapy with tucatinib; metformin coadministration	Pseudo-nephrotoxicity: serum creatinine rise without change in iohexol-measured GFR

Abbreviations: IV, intravenous; CT, computed tomography; AKI, acute kidney injury; CKD, chronic kidney disease; PC-AKI, post-contrast acute kidney injury; CI-AKI, contrast-induced acute kidney injury; eGFR, estimated glomerular filtration rate; GFR, glomerular filtration rate.

**Table 2:** Characteristics of the studies included in the hypersensitivity and acute adverse reactions section.

Study	Evidence Type	Oncology Setting	Treatment Context	Contrast-Related Issue
**General hypersensitivity framework**				
Brockow [62]	Narrative review/practice-oriented review	General clinical population; applicable to oncology risk interpretation	Diagnostic exposure to iodinated contrast media	Immediate versus nonimmediate reactions, clinical phenotypes, skin testing, cross-reactivity, and diagnostic work-up
Christiansen et al. [65]	Narrative review	General clinical population; relevant to oncology risk interpretation	Exposure to nonionic iodinated contrast media	Delayed cutaneous reactions, latency, risk factors, and T-cell-associated immunologic mechanisms
Suh et al. [11]	Systematic review and meta-analysis	General clinical population; applicable to oncology imaging	Intravenous administration of modern nonionic iodinated contrast agents	Pooled incidence of acute and severe reactions and comparison across different nonionic agents
Li et al. [10]	Retrospective clinical cohort	Mixed clinical population including patients with comorbid disease	Enhanced CT examinations with nonionic iodinated contrast media	Incidence of acute adverse reactions and patient-level risk factors, including asthma, cardiac insufficiency, prior reaction, and injection parameters
Idée et al. [63]	Critical narrative review	General clinical population; oncology-relevant because of IL-2 and beta-blocker discussion	Diagnostic exposure to iodinated contrast media	Mechanistic heterogeneity, rarity of severe/fatal reactions, delayed reactions, and inconsistent evidence for prophylaxis
Böhm et al. [69]	Retrospective clinical cohort	General radiology population; directly relevant to oncology imaging practice	Patients labeled as having iodine allergy undergoing CT evaluation	Inaccurate allergy labeling, poorer documentation, increased use of unenhanced CT, and consequences for clinical decision-making
Palmiere et al. [70]	Narrative/forensic review	General clinical population	Exposure to iodinated contrast media	Severe and fatal hypersensitivity reactions, unpredictability of catastrophic events, and post-mortem markers including mast cell tryptase and total IgE
**Oncology-specific immune modulation**				
Ridolfi et al. [51]	Retrospective clinical cohort	Mixed oncology population	Contrast-enhanced CT after treatment with ipilimumab, cytokines, chemotherapy, or targeted therapy	Higher incidence of immediate allergic-like reactions in patients receiving immune-modulating therapies, particularly CTLA-4 blockade
Hammond et al. [66]	Case report with translational immunologic work-up	Metastatic cancer/immune checkpoint inhibitor setting	Repeated contrast-enhanced CT after atezolizumab exposure	Severe delayed T-cell-mediated hypersensitivity after prior contrast tolerance, suggesting altered immune tolerance in the checkpoint inhibitor era
Zmolik et al. [71]	Case report	Thoracic oncology/immune checkpoint inhibitor setting	Contrast-enhanced CT during pembrolizumab treatment	Rare severe phenotype of contrast hypersensitivity presenting as allergic acute coronary syndrome with coronary vasospasm

Abbreviations: CT, computed tomography; IL-2, interleukin-2; IgE, immunoglobulin E; CTLA-4, cytotoxic T-lymphocyte-associated protein 4.

**Table 3:** Characteristics of the studies included in the thyroid dysfunction and radioiodine-related implications section on iodinated contrast media in oncology treatment settings.

Study	Evidence Type	Oncology Setting	Treatment Context	Contrast-Related Issue
Fang et al. [72]	Clinical cohort	Postoperative differentiated thyroid cancer	Planning postoperative radioiodine therapy after contrast-enhanced CT	Urinary iodine kinetics after iodinated contrast exposure in the radioiodine pathway
Vassaux et al. [73]	Mechanistic translational study; preclinical/translational	Thyroid iodide uptake with relevance to differentiated thyroid cancer	Radioiodine diagnostic and therapeutic pathway	Thyroid-selective impairment of iodide uptake and reduced NIS expression after iodinated contrast
Mishra et al. [74]	Prospective controlled study; clinical cohort	Differentiated thyroid carcinoma	Preoperative imaging before postoperative radioiodine ablation	Short-term urinary iodine increase after contrast-enhanced CT without long-term iodine retention
Peng et al. [23]	Prospective clinical cohort	General adult population	Intravenous iodinated contrast during routine imaging	Short-term thyroid-function changes, urinary iodine peak, and transient subclinical dysfunction after contrast exposure
Leidig-Bruckner [75]	Narrative/practice-oriented review	Thyroid disorders broadly, with relevance to thyroid cancer pathways	Radioiodine diagnostics and therapy; prophylaxis and monitoring in high-risk patients	Risk stratification, prevention, and management of iodinated-contrast-induced thyroid dysfunction

**Table 4:** Characteristics of the studies included in the drug interactions, isotope studies, and laboratory confounding section.

Study	Evidence Type	Oncology Setting	Treatment Context	Contrast-Related Issue
**Drug interactions, isotope studies, and renal-function confounding**				
Morcos et al. [18]	ESUR-based narrative/practice review	General clinical population; highly relevant to oncology because of polypharmacy and isotope-testing implications	Intravascular contrast administration during imaging and interventional procedures	Drug interactions, isotope-study interference, biochemical assay interference, and incompatibility of mixing contrast media with other agents
Hiremath et al. [80]	Systematic review protocol	General clinical population; relevant to oncology patients with CKD, diabetes, or cardiovascular disease	Peri-contrast management of metformin, RAS blockers, NSAIDs, and diuretics	Uncertainty regarding which drugs should be withheld before contrast procedures and when they should be restarted
van Leeuwen et al. [7]	Cross-sectional prevalence study	Ambulatory cancer patients receiving intravenous anticancer therapy	Polypharmacy during systemic cancer treatment	Background burden of potential drug interactions complicating attribution and management of contrast-related adverse events
Topletz-Erickson et al. [48]	Phase 1 clinical pharmacology study plus in vitro transporter work	HER2-positive solid-tumor drug-development context	Targeted therapy with tucatinib; metformin coadministration	Pseudo-nephrotoxicity and creatinine-based laboratory confounding without true decline in iohexol-measured GFR
Joshi et al. [81]	Preclinical/translational pharmacokinetic study	Carboplatin-based oncology dosing context	Iohexol used during chemotherapy-related renal-function assessment	Drug–drug interaction potential of iohexol as a renal filtration marker during anticancer therapy
**Laboratory assay interference**				
Otnes et al. [82]	Analytical/laboratory interference study	General clinical laboratory setting; relevant to oncology because of frequent biochemical monitoring	Contrast-agent contamination of biochemical samples and in vitro assay conditions	Analytical interference by contrast agents in biochemical assays, with potential for spurious laboratory results
Lippi et al. [83]	Narrative/laboratory medicine review	General clinical laboratory setting; highly relevant to oncology supportive care and treatment monitoring	Laboratory testing after administration of medical contrast media	Mechanisms and patterns of contrast-media interference with laboratory testing, including preanalytical and analytical confounding
Park et al. [84]	Experimental laboratory analyzer comparison study	General clinical laboratory setting; relevant to oncology because of routine chemistry follow-up	Effects of two medical contrast media on routine chemistry assays across automated analyzers	Analyzer-dependent effects of contrast media on routine chemistry results, supporting caution when interpreting post-contrast laboratory values

Abbreviations: ESUR, European Society of Urogenital Radiology; CKD, chronic kidney disease; RAS, renin–angiotensin system; NSAIDs, nonsteroidal anti-inflammatory drugs; HER2, human epidermal growth factor receptor 2; GFR, glomerular filtration rate.

**Table 5:** Practical preventive measures before iodinated contrast-enhanced CT in oncology patients.

Risk Domain/References	Examples of Higher-Risk Features	Preventive Measures	Follow-Up/Practical Notes
Renal vulnerability/PC-AKI risk [1,44,49,50]	eGFR < 30 mL/min/1.73 m^2^; AKI; recent renal deterioration; CKD; dehydration; sepsis or acute infection; heart failure; older age; repeated recent contrast exposure	Assess kidney function when clinically indicated; avoid unnecessary dehydration or prolonged fasting; use oral or intravenous hydration according to risk; use the lowest contrast dose compatible with diagnostic image quality; consider nephrology input in selected very-high-risk patients	Consider serum creatinine reassessment at 48–72 h in selected high-risk patients; evaluate alternative causes of AKI if renal function worsens
Nephrotoxic co-medication [1,18,37,80]	Cisplatin or other nephrotoxic anticancer drugs; aminoglycosides; NSAIDs; diuretics; selected targeted therapies; other nephrotoxic supportive-care drugs	Review medication list before imaging; temporarily withhold nephrotoxic or volume-depleting drugs only when clinically appropriate; avoid automatic discontinuation of essential treatment; coordinate with oncology/nephrology when needed	Restart withheld drugs when clinically safe; monitor renal function in patients with CKD, AKI, dehydration, or ongoing nephrotoxic exposure
High risk of immediate hypersensitivity reaction [11,49,56,57,62]	Previous immediate reaction to iodinated contrast; prior severe reaction; asthma or severe atopy; unclear iodine allergy label; beta-blocker use in selected patients	Obtain detailed allergy history; document culprit agent and reaction phenotype; avoid nonspecific iodine allergy labeling; consider alternative iodinated contrast agent when appropriate; consider allergy consultation or skin testing in selected patients; corticosteroid and/or antihistamine premedication may be used in selected high-risk patients according to local protocols	Ensure readiness to treat acute reactions; observe selected high-risk patients after injection; document any breakthrough reaction and the agent used
Delayed hypersensitivity reaction risk [54,57,58,59,61]	Previous delayed cutaneous reaction; severe delayed reaction; immune-modulating therapy; unclear timing or phenotype of prior reaction	Clarify timing, morphology, severity, and suspected culprit agent; consider dermatology/allergy evaluation, patch testing, or delayed-reading intradermal testing when appropriate; avoid re-exposure to the culprit agent after severe delayed reactions; consider alternative agent based on specialist input	Advise patients to report delayed rash or systemic symptoms after discharge; document delayed reactions carefully because they may occur after the imaging visit
Thyroid/radioiodine pathway [25,49,73,74]	Thyroid autonomy; nodular thyroid disease; differentiated thyroid carcinoma; planned thyroid scintigraphy or radioiodine therapy	Assess whether contrast-enhanced CT may interfere with planned radioiodine-based diagnosis or treatment; coordinate timing with endocrinology/nuclear medicine; consider thyroid-function monitoring in selected high-risk patients	Document recent iodine exposure; individualize timing of scintigraphy or radioiodine therapy after contrast exposure
Drug, isotope-study, and laboratory-test interference [18,82,83,84]	Polypharmacy; planned isotope studies; biochemical tests shortly after contrast; renal impairment delaying contrast clearance	Schedule non-urgent biochemical tests before contrast or delay them when feasible; communicate recent contrast exposure to laboratory and nuclear medicine teams; avoid mixing contrast media with incompatible drugs or solutions	Interpret unexpected post-contrast laboratory abnormalities cautiously, especially soon after contrast administration or in renal impairment
Frailty, poor oral intake, or environmental dehydration risk [31,44,49,50]	Older age; cachexia; vomiting; diarrhea; poor oral intake; hot season; limited access to fluids; outpatient status with limited monitoring	Avoid unnecessary fasting; provide clear instructions on permitted oral fluids; consider supervised oral or intravenous hydration in selected patients; adapt scheduling and preparation to local climate and patient frailty	Consider risk-adapted follow-up instructions for outpatients; advise patients to seek care for reduced urine output, persistent vomiting, or symptoms of dehydration

Abbreviations: AKI, acute kidney injury; CKD, chronic kidney disease; CT, computed tomography; eGFR, estimated glomerular filtration rate; NSAIDs, nonsteroidal anti-inflammatory drugs; PC-AKI, post-contrast acute kidney injury.

## Data Availability

No new data were created or analyzed in this study. Data sharing is not applicable to this article.

## Abbreviations

The following abbreviations are used in this manuscript:

AKI	Acute Kidney Injury
CI-AKI	Contrast-Induced Acute Kidney Injury
CIN	Contrast-Induced Nephropathy
CKD	Chronic Kidney Disease
CT	Computed Tomography
CTLA-4	Cytotoxic T-Lymphocyte-Associated Protein 4
DDI	Drug–Drug Interaction
DTC	Differentiated Thyroid Carcinoma
eGFR	Estimated Glomerular Filtration Rate
ESUR	European Society of Urogenital Radiology
HER2	Human Epidermal Growth Factor Receptor 2
IFN-γ	Interferon-gamma
NIS	Sodium-Iodide Symporter
NSAIDs	Nonsteroidal Anti-Inflammatory Drugs
PC-AKI	Post-Contrast Acute Kidney Injury
RAS	Renin–Angiotensin System
